# Structural characterization and biophysical analysis of recombinant SpoIVB variants: insights into PDZ and serine protease domain interactions

**DOI:** 10.1128/spectrum.00398-25

**Published:** 2025-08-14

**Authors:** Jian Zhu, Xu Zhang, Xinyun Zhang, Gaohui Sun, Peng Xu, Cai Yuan, Mingdong Huang, Longguang Jiang

**Affiliations:** 1 College of Chemistry, Fuzhou University 12423, Fuzhou, Fujian, China; 2 School of Life Sciences, Yunnan University, Kunming, Yunnan, China; 3 College of Biological Science and Engineering, Fuzhou University, Fuzhou, Fujian, China; 4 National and Local Joint Engineering Research Center on Biopharmaceutical and Photodynamic Therapy Technologies, Fuzhou University, Fuzhou, Fujian, China; Rowan University Cooper Medical School, Camden, New Jersey, USA

**Keywords:** *Bacillus subtilis*, SpoIVB, crystal structure, SAXS

## Abstract

Sporulation factor IV B protease (SpoIVB), as a PDZ-protease, plays a central role in cellular differentiation via activating pro-σ^K^ processing at the σ^K^ checkpoint during spore formation. However, the molecular mechanism and structure of SpoIVB remain unclear. In this study, we expressed and characterized several recombinant variants of SpoIVB, including SpoIVB_75-426_, SpoIVB_75-426-S378A_, and SpoIVB_101-426-S378A_. Their structural properties were analyzed through dynamic light scattering, size-exclusion chromatography, small-angle X-ray scattering (SAXS), and X-ray crystallography. The crystal structure of SpoIVB_101-426-S378A_ was determined at 2.49 Å resolution, revealing a unique PDZ domain arrangement and an unusual catalytic triad in the serine protease domain. SAXS analysis demonstrated that SpoIVB_75-426-S378A_ adopts a monomeric form with a folded but flexible structure in solution, while the S378A mutation alters its hydrodynamic radius (*R*_*H*_) and overall compactness. These findings provide new insights into the structural dynamics of SpoIVB, including its monomeric state, PDZ domain interactions, and the functional implications of the S378A mutation. This study lays the groundwork for further investigations into the mechanistic role of SpoIVB in biological systems and its potential as a therapeutic target.

**IMPORTANCE:**

Sporulation factor IV B protease (SpoIVB) is a pivotal PDZ-protease regulating the σ^K^ checkpoint during bacterial sporulation, yet its structural and mechanistic details remain elusive. This study provides the first atomic-resolution crystal structure of a SpoIVB variant (SpoIVB_101-426-S378A_), revealing a non-canonical catalytic triad (His236-Ala378-Thr393) and a unique PDZ domain insertion into the serine protease core. Biophysical analyses demonstrate that the S378A mutation enhances structural compactness and monomeric stability, while small-angle X-ray scattering confirms flexibility in the N-terminal region. These findings challenge traditional views of serine protease mechanisms and unveil novel regulatory interactions between PDZ and catalytic domains. The structural insights advance understanding of SpoIVB’s role in σ^K^ activation and lay a foundation for targeting PDZ-protease interfaces in antibacterial strategies. This work bridges critical gaps in bacterial developmental biology and highlights SpoIVB as a potential therapeutic target.

## INTRODUCTION

Sporulation, a sophisticated process through which certain bacteria transition into dormant spores to withstand harsh environmental conditions, exemplifies their evolutionary adaptability and resilience (1–4). This intricate developmental pathway ensures the long-term survival of bacterial species by enabling them to withstand extremes of temperature, nutrient deprivation, and other environmental stresses that would otherwise be lethal in their vegetative state (5, 6). Central to the sporulation process in *Bacillus subtilis* and related species is the sigma-K checkpoint, a pivotal regulatory stage that orchestrates the final stages of spore formation with meticulous precision (7, 8).

At the heart of the sigma-K checkpoint lies sporulation factor IV B protease (SpoIVB), a peptidase enzyme critical for activating pro-σ^K^, a transcription factor essential for late sporulation gene expression (9–11). SpoIVB is a serine protease of a new family of trypsin-like enzymes belonging to the clan PA (S55) (12). SpoIVB belongs to the widespread family of PDZ-proteases that combine a PDZ domain and a serine protease domain (12–16). PDZ domains are modular protein-protein interaction modules that typically bind to short linear motifs. They play critical roles in organizing signaling complexes, scaffolding subcellular structures, and regulating enzymatic activity. PDZ domains are characterized by a conserved β-sandwich fold comprising six β-strands and two α-helices (17). In proteases, PDZ domains often serve as regulatory elements by recruiting substrates or modulating catalytic activity through conformational changes (18).

SpoIVB initially exists in an inactive zymogen form, requiring precise regulation to prevent premature activation (19, 20). Previous studies have proposed this regulation of autoproteolysis of SpoIVB through BofC (21, 22). In this process, mature SpoIVB binds to the C-terminus of BofA, resulting in the cleavage of SpoIVFA’s C-terminus by SpoIVB. Ultimately, SpoIVFB is released from the ternary complex to activate pro-σ^K^ into σ^K^ (23, 24).

Despite prior structural studies on SpoIVB and BofC (16, 25, 26), the atomic-resolution architecture of SpoIVB, particularly in complexes with regulatory proteins like BofC or SpoIVFA, remains unresolved. This gap impedes the understanding of its activation mechanism and regulatory interactions. Understanding the atomic-resolution structure of SpoIVB is essential for deciphering its activation mechanisms and how BofC modulates its function. This gap in knowledge highlights an ongoing challenge in the field of bacterial developmental biology, underscoring the need for detailed structural studies to complement functional analyses.

In this study, we present structural and biophysical analyses of several recombinant variants of the SpoIVB protein, including SpoIVB_75-426_, SpoIVB_75-426-S378A_, and SpoIVB_101-426-S378A_. Through a combination of size-exclusion chromatography (SEC), dynamic light scattering (DLS), small-angle X-ray scattering (SAXS), and X-ray crystallography, we have gained significant insights into the structural dynamics, catalytic mechanisms, and monomeric state of SpoIVB. Our work provides novel structural information on the PDZ and serine protease domains of SpoIVB, including an unexpected rearrangement of the catalytic triad and unique domain interactions. These findings contribute to a deeper understanding of the functions of SpoIVB and its potential as a therapeutic target in PDZ-protease-related biological processes.

## MATERIALS AND METHODS

### Constructions of SpoIVB variants expression vectors

The gene encoding *B. subtilis* truncated SpoIVB (Thr75-Ser426) was amplified by PCR as described previously (25). The *SpoIVB_75-426_* was cloned into the pCold I vector by the LIC method to generate SpoIVB_75-426_ with a 6×His tag at the N-terminus. The sequencing confirmed that the pCold I-SpoIVB_75-426_ plasmid was successfully constructed. Using the same method, we constructed the other plasmid of the truncated SpoIVB_101-426_ (Leu101-Ser426) in the pCold I vector (pCold I-SpoIVB_101-426_). Each construct was transformed into a competent *Escherichia coli* (DH5_α_) and subsequently screened on Luria Broth (LB) agar plates (composed of 0.5% yeast extract, 1% tryptone, 1% NaCl, and 2% agar) containing 100 µg/mL ampicillin to identify positive colonies. To achieve a stable recombinant SpoIVB, we performed site-directed mutagenesis to mutate Ser378 to Ala (SpoIVB_75-426-S378A_ and SpoIVB_101-426-S378A_, respectively). All recombinant SpoIVB expression plasmids were verified through sequencing.

### Expression and purification of recombinant SpoIVB variants

*E. coli* BL21(DE3) was transformed with expression plasmids, and a single colony was inoculated into 100 mL LB medium with 100 µg/mL ampicillin and grown overnight at 37°C. After 12 h, the starter culture was utilized to inoculate 1 L of medium containing 100 µg/mL ampicillin. The cultures were grown at 37°C with agitation at 200 rpm to an OD_600_ = 0.6–0.8. After cooling the culture to 16°C, it was induced with 0.1 mM isopropyl β-D-thiogalactoside and incubated at 16°C and 180 rpm for 12 h in a shaker. The bacterial pellets were then stored at −80°C for further use.

Centrifugation of the induced bacterial solution was carried out at 4,000 rpm for 20 min, followed by discarding the supernatant and resuspending the bacterial pellets in 30 mL lysis buffer containing 20 mM Tris (pH 7.4), 200 mM NaCl, and 5% glycerol. Resuspended cells were then lysed using a high-pressure cell disruptor at 800 bar, with slow pressurization and a crushing time of 3 min. The crushed solution was centrifuged at 18,000 rpm for 30 min, and the supernatant was filtered through 0.45 µm filter membranes and subsequently exchanged into an equilibrium buffer consisting of 20 mM Tris (pH 7.4), 200 mM NaCl, and 5% glycerol. Following this, recombinant SpoIVB variants were purified using Ni Sepharose Excel resin (GE, USA). Impurities were removed using a gradient elution with 10–50 mM imidazole. The target protein was then eluted with 10 mL of elution buffer consisting of 20 mM Tris (pH 7.4), 200 mM NaCl, 5% glycerol, and 300 mM imidazole. The eluted proteins were concentrated using a 10 kDa molecular weight cutoff spin concentrator and loaded onto a Superdex 200 Increase 10/300 GL column (GE, USA), which was equilibrated with a buffer consisting of 20 mM Tris (pH 7.4), 200 mM NaCl, and 5% glycerol. All fractions were analyzed on 15% sodium dodecyl sulfate polyacrylamide gel electrophoresis (SDS-PAGE) and were stained with Coomassie Brilliant Blue R-250. For crystal growth experiments, all the purified recombinant SpoIVB variants were concentrated at 15 mg/mL.

### Dynamic light scattering

DLS measurements were conducted using a Nano ZS ZEN 3600 Instrument (Malvern, UK), equipped with a 2 mL micro-sampling cell at 25°C. Each SpoIVB variant was diluted in a buffer consisting of 20 mM Tris (pH 7.4), 200 mM NaCl, and 5% glycerol to achieve protein concentrations of 0.10 mg/mL, 0.25 mg/mL, and 0.50 mg/mL. Before the DLS measurements, all solutions were filtered through a 0.22 µM Millipore filter membrane to eliminate dust particles. The cuvette was inserted into the instrument and allowed to equilibrate for 2 min at 25°C. The obtained data were analyzed using the Dynamics software package, version 5.

### Crystallization, data collection, and processing

We utilized a Phoenix crystallization robot (Art Robbins Instruments, Sunnyvale, CA, USA) alongside commercial screen kits from Qiagen (Hilden, Germany), XtalQuest (Beijing, China), and Hampton Research (Aliso Viejo, CA, USA) to screen for crystallization conditions of recombinant SpoIVB variants using the sitting-drop vapor-diffusion method. After 4 days at 25°C, reproducible crystals of SpoIVB_101-426-S378A_ were successfully obtained under the following conditions: 25% PEG3350, 0.2 M lithium sulfate, and 0.1 M HEPES at pH 7.5. Detailed information regarding these conditions is presented in Table S1. The crystals were harvested and stored in crystallization solutions supplemented with 25% vol/vol glycerin. Subsequently, these crystals were flash-frozen in liquid nitrogen to facilitate X-ray data collection.

Data were collected at 100 K under cryogenic conditions on beamline BL10U2 at the Shanghai Synchrotron Radiation Facility. All 360 images were acquired, with each image captured at a crystal-to-detector distance of 300 mm and an exposure time of 1 s per 1° oscillation frame. The collected data were processed using the HKL2000 package (27). A summary of the data collection and processing statistics is provided in Table 1.

**TABLE 1:** X-ray data collection and model refinement statistics for SpoIVB_101-426-S378A_ crystal^*a*^

Crystal	SpoIVB_101-426-S378A_
Data collection	
X-ray source wavelength (Å)	1.0
Resolution limits (Å)	50.00–2.49 (2.66–2.49)
Space group	P62
Temperature of experiments (K)	100
Cell constants	*a* = *b* = 72.190 Å, *c* = 108.440 Å, α = β = 90°, γ = 120°
Completeness (%)	100
Multiplicity	20.2
CC_1/2_	0.995
*R*_merge_^*b*^	0.117 (2.066)
Number of observations	209559
Number of unique reflections	11,228
Refinement data	
*R* factor	0.2356
*R*_free_	0.2874
Average B-factor (Å^2^) of protein	91.686
Root mean square deviation of bond lengths (Å)	0.010
Root mean square deviation of angle (°)	1.284
Ramachandran analysis (%)	92.38^*c*^, 6.71^*d*^, 0.91^*e*^

^*a*^ Note: the highest-resolution shell is shown in parentheses.

^*b*^ R_merge_ = Σ|*Ii – <I* > |/Σ*Ii*, where *Ii* is the intensity of the *i*th observation and *<I* > is the mean intensity of the reflections.

^*c*^ Percentage of residues in most favored regions.

^*d*^ Percentage of residues in additional allowed regions.

^*e*^ Percentage of residues in disallowed regions.

### Phasing and refinement

The structure of SpoIVB_101-426-S378A_ was determined using the molecular replacement method (28). The search model was a predicted structure of SpoIVB_101-426-S378A_ modified from the structure of the full length of apo-SpoIVB retrieved from the AlphaFold protein structure database (https://www.alphafold.ebi.ac.uk/) (29). The molecular replacement model underwent iterative refinement and manual model rebuilding using Refmac (30) and Coot (31), alternately. At a resolution range of 36.36–2.59 Å, the final refined model exhibited an *R* factor of 0.2356 and a *R*_free_ factor of 0.2874 (Table 1). The structure was validated using PROCHECK (32). The coordinate of the final model was deposited in the Protein Data Bank (PDB) with an access code of 9LNF. The results were analyzed and visualized using PyMol (33).

### SAXS data collection and analysis

The SAXS data were collected at the BL19U2 beamline at the National Facility for Protein Science Shanghai. All data sets were acquired with an exposure time of 1 second at a temperature of 283 K, using a wavelength of 1.033 Å. Measurements were conducted using three different protein concentrations: 1, 2, and 3.7 mg/mL. The 2D scattering images were converted to 1D SAXS curves using the BioXTAS RAW software package (34). Buffer scattering was subtracted from the sample scattering using PRIMUS (35). Linear analysis was performed in the Guinier region of the scattering data for all preparations. The pair distribution functions of the particles, *P_(r)_*, and the maximum sizes, *D*_max_, were calculated using the GNOM program (36).

Low-resolution shapes were determined from solution scattering data using DAMMIF, a program from the ATSAS software suite (37, 38). For each data set, 20 independent calculations were performed using default parameters and without symmetry constraints. These 20 reconstructions were then averaged and filtered to create a final consensus model using the DAMAVER suite (39). The final bead models were visualized and analyzed using Chimera (40) and PyMol (33). A summary of the data collection and final model refinement statistics is provided in Table S2.

## RESULTS

### Production and characterization of stable SpoIVB variants

**Fig 1:**
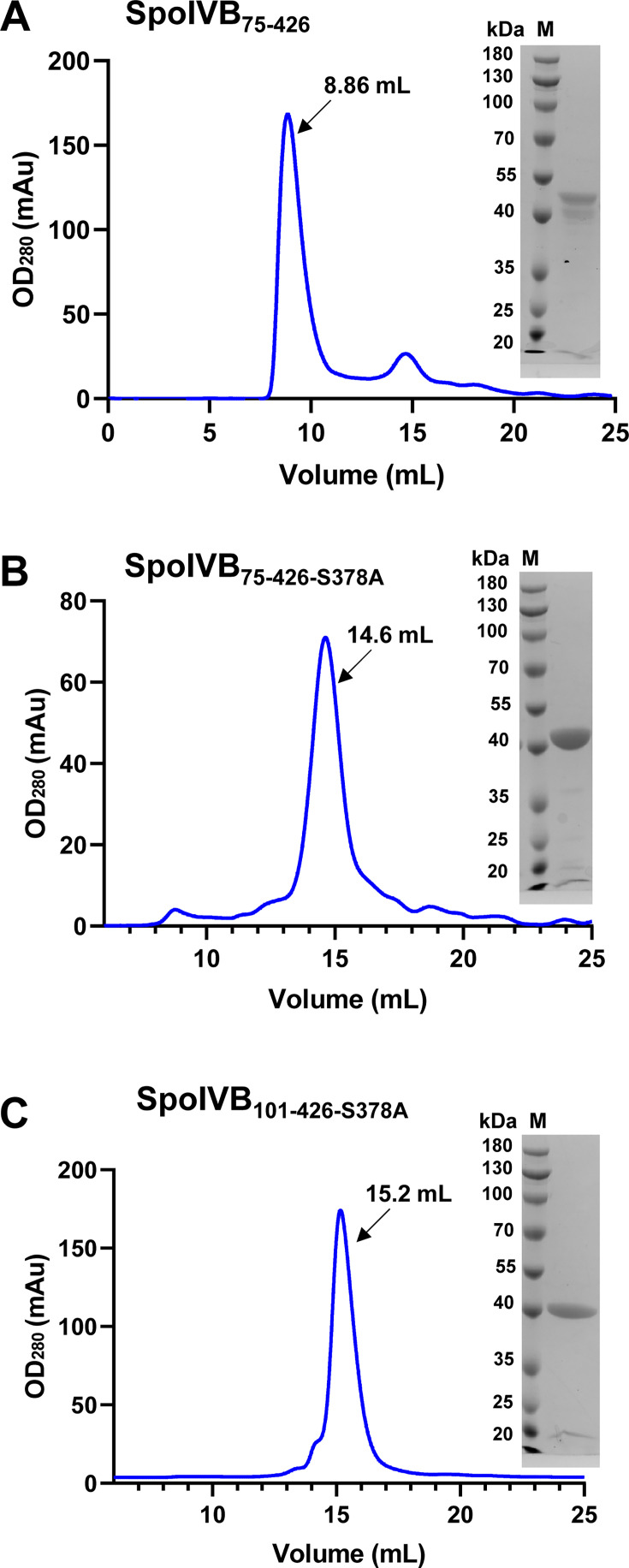
Purifications of SpoIVB variants by gel filtration chromatography (Superdex 200 Increase 10/300 GL) and confirmed by 15% SDS-PAGE. (**A**) SEC profile of SpoIVB_75-426_, inset: 15% SDS-PAGE with purified SpoIVB_75-426_. (**B**) SEC profile of SpoIVB_75-426-S378A_, inset: 15% SDS-PAGE with purified SpoIVB_75-426-S378A_. (**C**) SEC profile of SpoIVB_101-426-S378A,_ inset: 15% SDS-PAGE with purified SpoIVB_101-426-S378A_.

Although we previously generated a computational model of SpoIVB_75-426-S378A_ fused to modified maltose binding protein using SWISS-MODEL and docked it into a SAXS-derived molecular envelope (25), we still did not get a crystal of mMBP-SpoIVB_75-426-S378A_. Thus, we used the pCold I vector to express the recombinant SpoIVB_75-426_ (Fig. S1A) and SpoIVB_75-426-S378A_ (Fig. S1B) alone in the *E. coli* expression system. In addition, we analyzed the predicted structure of SpoIVB from AlphaFold and found the structure of the residues 75–100 at the N-terminal as a small and flexible domain that might decrease the possibility of crystallization (Fig. S2). Thus, we expressed the truncated and mutated SpoIVB, starting from the PDZ domain to the serine protease domain (Leu101 to Ser426), including SpoIVB_101-426_ and SpoIVB_101-426-S378A_, but only SpoIVB_101-426-S378A_ was expressed successfully (Fig. S1C). To further purify these recombinant SpoIVB variants, the elution fractions were subjected to a gel filtration column (Superdex 200 Increase 10/300 GL). The SEC profiles revealed that the elution peaks of SpoIVB variants are highly homogeneous (Fig. 1), and the elution volume (Ve) of SpoIVB_75-426_ (8.86 mL) is lower than those of SpoIVB_75-426-S378A_ (14.6 mL) and SpoIVB_101-426-S378A_ (15.2 mL) (Table 2). Subsequently, the elution fractions were collected and subjected to a 15% SDS-PAGE analysis, which confirmed the high purity of both recombinant SpoIVB variants (Fig. 1). Following this, recombinant SpoIVB variants were concentrated to facilitate further studies, including DLS, SAXS, and screening conditions conducive to crystal growth.

**TABLE 2:** Comparison of the Ve, hydrodynamic radius (*R*_*H*_), and molecular weight (MW) of SpoIVB variants

Variants	Ve (mL)	*R*_*H*_ (nm)	Theoretical MW (kDa)	SAXS MW (kDa)
SpoIVB_75-426_	8.86	16.87	39.1	N/A
SpoIVB_75-426-S378A_	14.6	4.95	39.1	38.1
SpoIVB_101-426-S378A_	15.2	4.75	36.8	N/A

### Hydrodynamic properties reveal mutation-induced structural compactness

**Fig 2:**
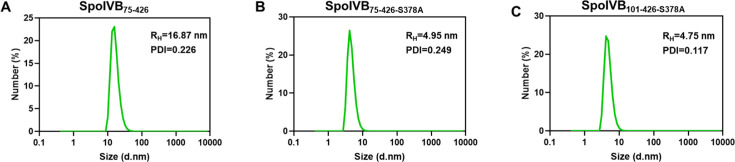
DLS analysis of SpoIVB variants. (**A**) Particle size distribution of SpoIVB_75-426_ is 16.87 nm. (**B**) Particle size distribution of SpoIVB_75-426-S378A_ is 4.95 nm. (**C**) Particle size distribution of SpoIVB_101-426-S378A_ is 4.75 nm.

Both chromatography and SDS-PAGE profiles of recombinant SpoIVB variants showed that the protein existed predominantly in a homogeneous form. Then, we used the DLS method to analyze the size distributions of SpoIVB variants (SpoIVB_75-426_, SpoIVB_75-426-S378A_, and SpoIVB_101-426-S378A_) at three different concentrations of 0.10, 0.25, and 0.50 mg/mL, respectively. The size distributions of SpoIVB variants at different concentrations are consistent and show a single species (Fig. S3), demonstrating that the size distribution of SpoIVB variants is unaffected by different concentrations. However, the values of hydrodynamic radius (*R*_*H*_) of SpoIVB variants are different, and the *R*_*H*_ of SpoIVB_75-426_ is 16.87 nm (Fig. 2A), whereas SpoIVB_75-426-S378A_ exhibited a significantly reduced *R*_*H*_ of 4.95 nm (Fig. 2B), consistent with the result in the gel filtration chromatography, in which the Ve of SpoIVB_75-426_ is smaller than that of SpoIVB_75-426-S378A_ (Table 2). The *R*_*H*_ of SpoIVB_101-426-S378A_ is 4.75 nm (Fig. 2C) and slightly smaller than that of SpoIVB_75-426-S378A_. This result consists of the molecular weight (MW) difference between these two proteins (Table 2). These results demonstrated that SpoIVB_75-426_, SpoIVB_75-426-S378A_, and SpoIVB_101-426-S378A_ are all monodispersed, but only SpoIVB_75-426-S378A_ and SpoIVB_101-426-S378A_ did not form a multimer.

### Crystal structure unveils an atypical PDZ-protease architecture

Although we tried to screen the crystal growth conditions of SpoIVB_75-426_, SpoIVB_75-426-S378A_, and SpoIVB_101-426-S378A_, we successfully obtained the crystals of SpoIVB_101-426-S378A_ in 25% PEG3350, 0.2 M lithium sulfate, and 0.1 M HEPES at pH 7.5 (Fig. S4). We determined the crystal structure of SpoIVB_101-426-S378A_ in the monoclinic space group *P*62 with one monomer in the asymmetric unit (ASU) at a resolution of 2.49 Å (Table 1). This study reports the first experimental atomic-resolution structure of a SpoIVB variant, resolving critical features of its PDZ-protease architecture. The ASU contains one SpoIVB_101-426-S378A_ molecule. In the overall structure, all residues of SpoIVB_101-426-S378A_ have been meticulously modeled into the electron density map. Additionally, the N-terminal motif, consisting of four residues (Arg-His-Met-Gly) derived from the pCold I plasmid, has also been incorporated into the electron density map.

**Fig 3:**
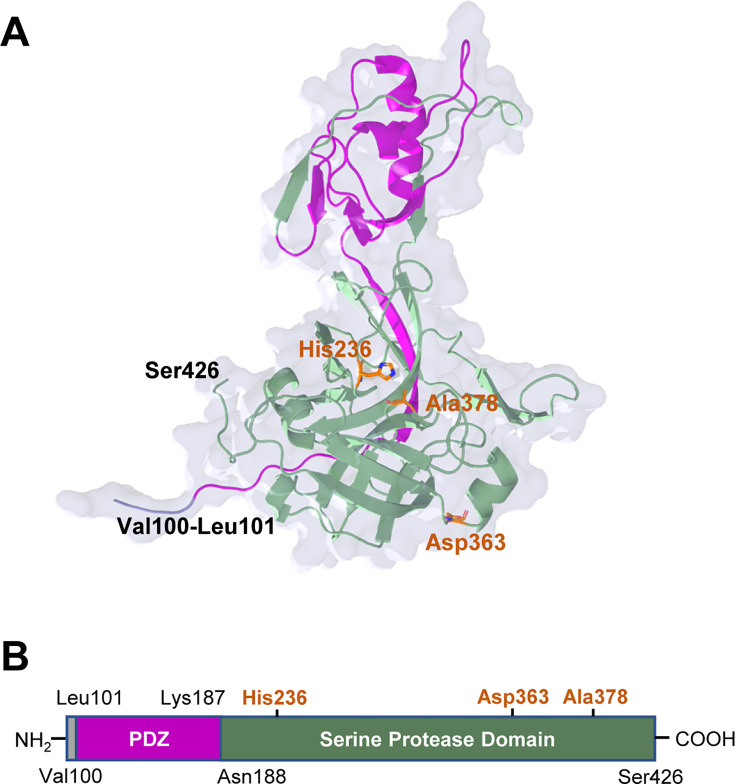
Structural architecture of SpoIVB_101-426-S378A_. (**A**) The ribbon model of SpoIVB_101-426-S378A_ is colored according to its PDZ and serine protease domains. Residues constituting the proteolytic site (His236, Asp363, and Ala378) are shown as stick models. (**B**) Domain organization of SpoIVB_101-426-S378A_ indicates the domain borders and catalytic residues.

**Fig 4:**
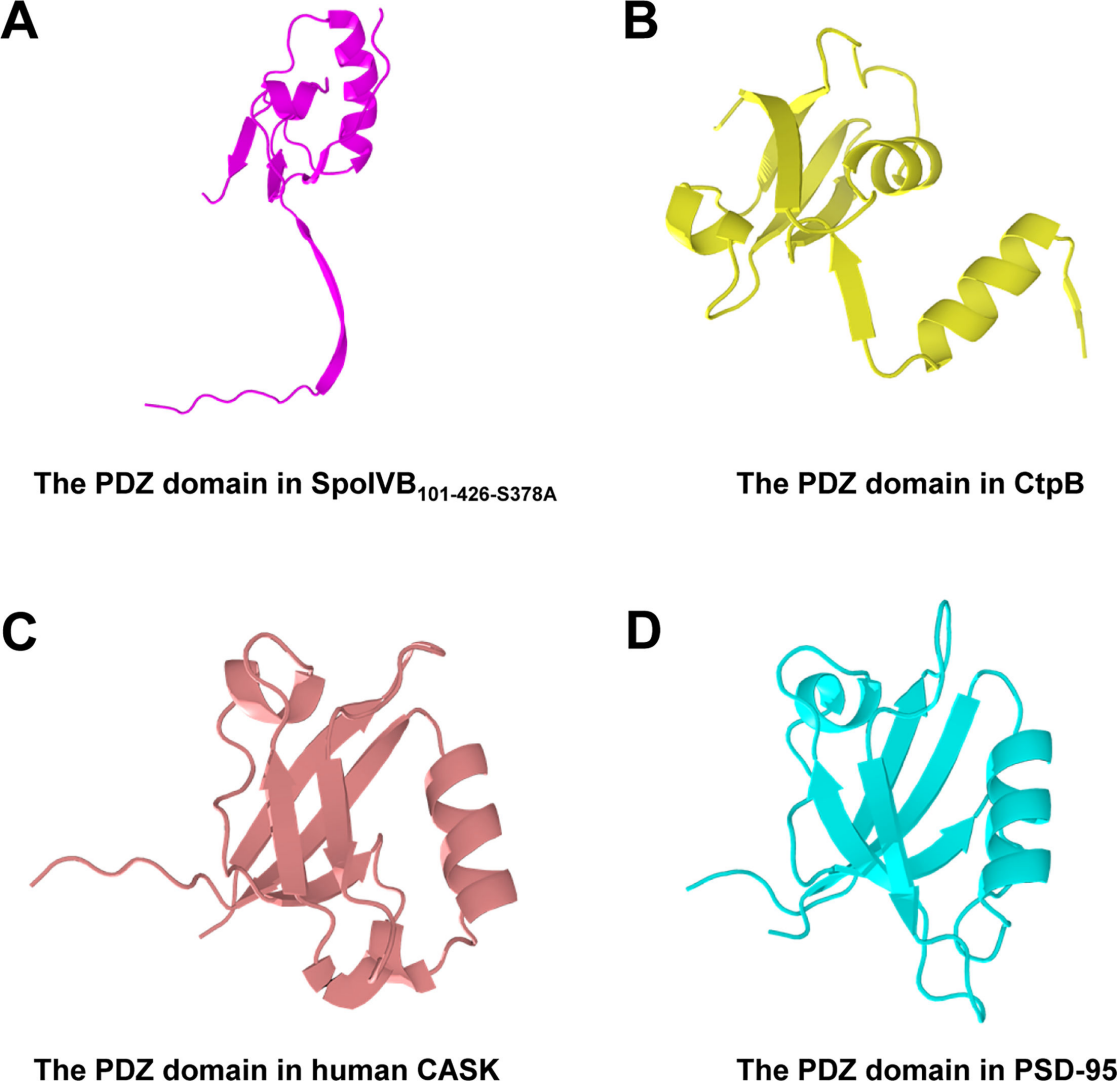
The structures of PDZ domains in different proteins. (**A**) The PDZ domain in SpoIVB_101-426-S378A_ has two α helices and four β strands, and one of the β strands looks like a tail inserted into the core of the serine protease domain. (**B**) The PDZ domain in CtpB (43). (**C**) The PDZ domain in human CASK (42). (**D**) The PDZ domain in PSD-95 (17).

SpoIVB_101-426-S378A_ comprises one PDZ domain and one SPD domain (Fig. 3). The classical PDZ domains consist of a compact arrangement of two α helices and six β strands; their structures have been solved in previous studies (17, 41–43). However, the structure of the PDZ domain in SpoIVB_101-426-S378A_ is different from other reported PDZ domains in other proteins, including carboxy-terminal processing protease (CtpB), calcium/calmodulin-dependent serine protein kinase (CASK), and postsynaptic density protein-95 (PSD-95) (Fig. 4). The PDZ domain in SpoIVB_101-426-S378A_ has two α helices and four β strands, and one of the β strands looks like a tail inserted into the core of the serine protease domain (Fig. 3A). The PDZ domain in SpoIVB belongs to PDZ class I, which binds to the PDZ-binding motif (Thr-His-Val) in the C-terminal of the other SpoIVB molecule (44). In the crystal structure of SpoIVB_101-426-S378A_, we observe that the N-terminal before the PDZ domain of one symmetrical SpoIVB_101-426-S378A_ molecule, but not the PDZ domain itself, inserts into the other SpoIVB_101-426-S378A_ molecule and binds to the PDZ-binding motif (Thr393-His394-Val395), suggesting that regions outside the canonical PDZ domain (e.g., the N-terminal loop) may engage with PDZ-binding motifs (Fig. S5). While likely non-physiological, this suggests regions beyond the PDZ domain may contribute to partner recognition.

**Fig 5:**
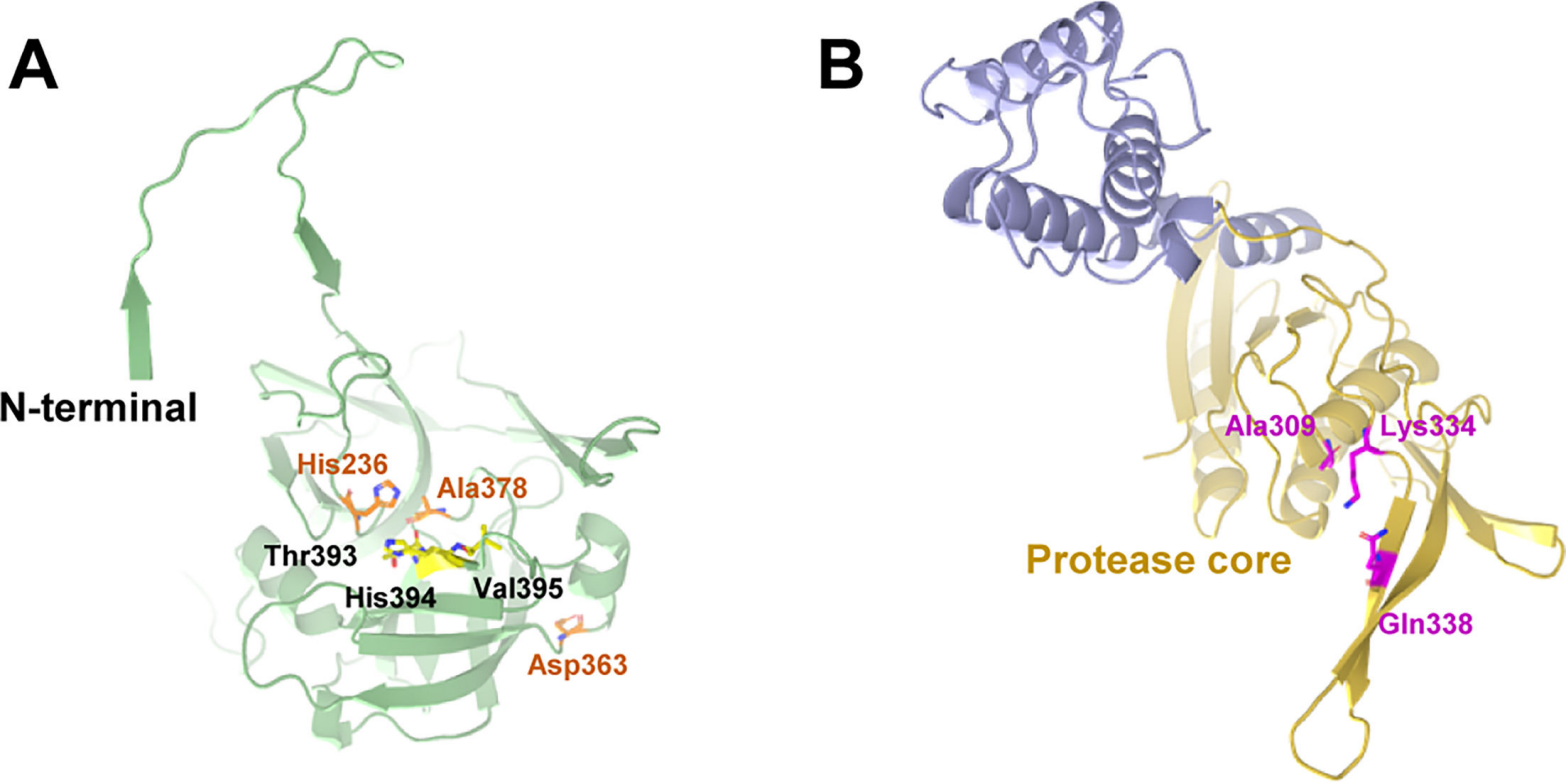
The structures of the serine protease domain in SpoIVB and CtpB. (**A**) The serine protease domain in SpoIVB_101-426-S378A_ has a long N-terminal structure (Glu188-Ser214) that swaps into the PDZ domain. Moreover, the catalytic triad in SpoIVB is not a canonical arrangement, in which the position of Asp363 is far away from His236 and Ala378. (**B**) The catalytic triad in CtpB is a canonical arrangement and comes closer to the tertiary structure to form the active site of CtpB.

Although SpoIVB and CtpB both belong to the ubiquitous family of PDZ-proteases, which feature a combination of a catalytic SPD domain and a regulatory PDZ domain, the SPD structure in SpoIVB is notably unique. Firstly, the SPD structure in SpoIVB not only has a distinctive spherical structure consisting of two beta-barrel sub-domains that converge at the catalytic active site but also has a lengthy N-terminal structure (Glu188-Ser214) that swaps into the PDZ domain (Fig. 5A). Secondly, the spatial arrangement of the catalytic triad in SpoIVB is different from the canonical arrangement, and His236 (His57 in chymotrypsin numbering) is close to Ala378 (Ala195 in chymotrypsin numbering); however, the atypical spatial arrangement of His236 and Ala378, with Asp363 (Asp102 in chymotrypsin numbering) displaced (~24 Å from His236), suggests a divergent catalytic mechanism. We hypothesize that Thr393 may compensate for Asp363’s absence, potentially acting as a nucleophile. Compared with that, the catalytic triad in inactive CtpB is still in the canonical arrangement and comes closer to the tertiary structure to form the active site of CtpB (Fig. 5B). The above structural information hints that Asp363 in SpoIVB might not be a catalytic residue; moreover, the position of Thr393 is more likely a third catalytic residue.

### SAXS confirms conformational flexibility of SpoIVB_75-426-S378A_ in solution

**Fig 6:**
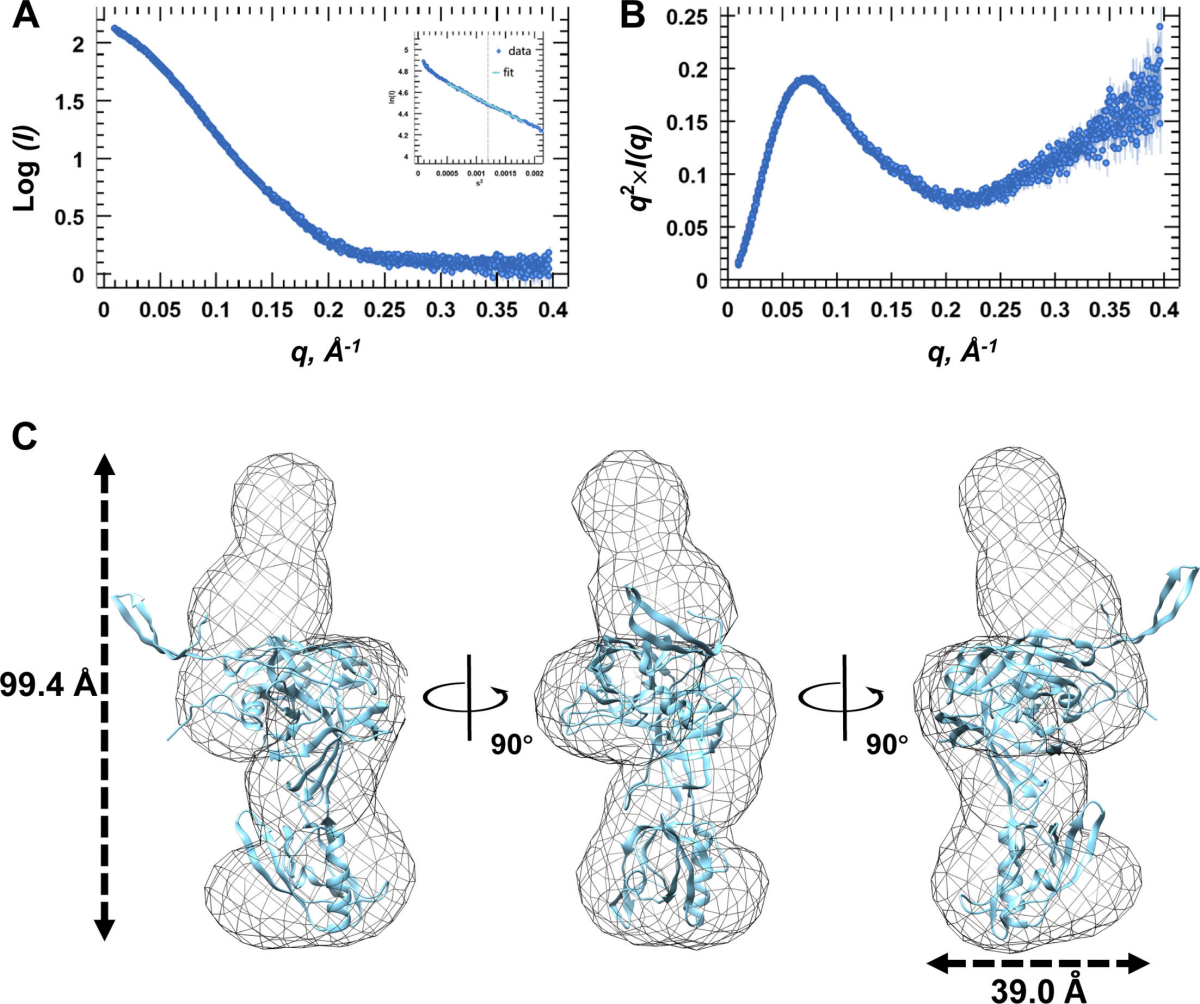
SAXS analyses of SpoIVB_75-426-S378A_. (**A**) Experimental SAXS scatter plot of SpoIVB_75-426-S378A_ in solution. The Guinier region and the corresponding linear fitting are shown in the inset. (**B**) Kratky plot calculated from the experimental data. (**C**) Superimposition of the BioSAXS envelope (shown as a gray mesh) onto SpoIVB_75-426-S378A_ (shown in cyan) represented as a cartoon.

Although we did not obtain crystals of SpoIVB_75-426-S378A_, we used the biological SAXS (BioSAXS) experiment to investigate its structure in solution across various concentrations (1.0, 2.0, and 3.7 mg/mL). Notably, no unspecific aggregation of SpoIVB_75-426-S378A_ was observed at the highest concentration (3.7 mg/mL), prompting us to utilize this concentration for BioSAXS shape reconstruction and modeling (Fig. 6A). Kratky analysis was employed to assess the protein’s folding degree, revealing a peak at low *q* values followed by an increase at high *q* values, indicating SpoIVB_75-426-S378A_ being folded yet somewhat flexible in solution (Fig. 6B).

The radius of gyration (*R_g_*) of SpoIVB_75-426-S378A_ was determined to be 29.76 ± 0.18 Å. Using SAXSMoW with an integration range constrained to qR*_g_* <1.3 and *q*_max_ limited to *I(0)*/*I(q_m_)* = 10^2:25^, the MW of SpoIVB_75-426-S378A_ was calculated to be 38.1 kDa (Table S2). This value aligns closely with the MW derived from the amino acid sequence (39.1 kDa), confirming that SpoIVB_75-426-S378A_ exists as a monomer, in accordance with the results obtained from SEC and DLS measurements.

Although the structure of full-length SpoIVB is unknown, we downloaded the predicted three-dimensional structure of SpoIVB (AF-P17896-F1) from the AlphaFold server. Using the program Chimera, we used the truncated SpoIVB_75-426-S378A_ as a model to be fitted into the molecular envelope of SAXS (Fig. 6C). In this model, the N-terminal part of SpoIVB_75-426-S378A_ cannot be fitted into the envelope, indicating the orientation of this part might be flexible and different from that predicted by AlphaFold. Additionally, the flexible N-terminal region (residues 75–100) likely introduces conformational heterogeneity, explaining the failure to crystallize full-length SpoIVB.

## DISCUSSION

The expression, purification, and structural characterization of recombinant SpoIVB variants have provided significant insights into its biophysical properties, the structural elements critical for its function, and its potential to interact with other molecules. There are several self-cleavage sites, including Val52-|-Asn53, Ala62-|-Phe63, and Val74-|-Thr75 at the N-terminus of SpoIVB. The truncation at Leu101 was guided by AlphaFold prediction. In addition, the S378A mutation was introduced to eliminate proteolytic activity, thereby preventing auto-degradation during expression and purification. Thus, our study has focused on the truncated variants of SpoIVB, including SpoIVB_75-426_, SpoIVB_75-426-S378A_, and SpoIVB_101-426-S378A_, which were expressed in *E. coli* and analyzed using several biophysical techniques, including SEC, DLS, SAXS, and X-ray crystallography.

The successful expression of SpoIVB_101-426-S378A_ marks significant progress, particularly given the challenges encountered with the crystallization of the full-length protein. Our results show that while SpoIVB_75-426_ failed to crystallize, the truncated SpoIVB_101-426-S378A_ could be produced in high yields and with high purity, indicating the truncation might relieve structural constraints that impede crystallization. The differential elution profiles observed in SEC indicate that the S378A mutation leads to subtle conformational changes that affect the overall size and shape of the protein. Specifically, SpoIVB_75-426_ eluted at a lower volume than the other variants, which is consistent with the observed increase in the *R*_*H*_ for this variant. The S378A mutation prevents autoproteolysis, stabilizing SpoIVB in a monomeric state. While SAXS confirms flexibility in the N-terminus, the SEC and DLS profiles primarily reflect the transition from aggregated (wild-type) to monomeric (mutant) states, rather than structural rigidity. The aggregation of SpoIVB_75-426_ may reflect structural instability rather than functional oligomerization. Future studies will clarify whether multimerization is physiologically relevant or an artifact of autoproteolysis.

DLS analysis demonstrated that all recombinant SpoIVB variants exist as monodispersed species in solution, with the S378A mutation leading to a reduced *R*_*H*_, supporting the findings from SEC. DLS measurements revealed a significant difference in the *R*_*H*_ of SpoIVB_75-426_ compared to SpoIVB_75-426-S378A_, which suggests that the mutation at position 378 may induce a more compact and rigid structure. Thus, the SEC and DLS data indicate that the S378A mutation prevents aggregation, enabling isolation of monomeric SpoIVB. This is further supported by SAXS data, where SpoIVB_75-426-S378A_ showed a clear scattering profile indicative of a monomeric structure. The obtained MW from SAXS analysis closely matched the theoretical value based on the amino acid sequence, confirming that SpoIVB_75-426-S378A_ exists primarily as a monomer in solution.

Kratky analysis from the SAXS data indicated that SpoIVB_75-426-S378A_ is folded but exhibits some degree of flexibility. The scattering profile suggests that while the protein adopts a well-defined structure, its N-terminal domain may remain flexible, which agrees with the molecular envelope analysis. These observations are crucial for understanding the dynamic behavior of SpoIVB in solution, particularly how its flexibility might impact its interactions with other proteins or cellular components.

The high-resolution crystal structure of SpoIVB_101-426-S378A_ at 2.49 Å revealed novel structural features that distinguish it from other PDZ-proteases. The structure confirmed that the PDZ domain of SpoIVB is part of a unique architecture with a tail-like extension (β3, residues 142–148) that inserts into the core of the serine protease domain, suggesting a potentially new mode of PDZ domain regulation. Unlike canonical PDZ architectures, this unique arrangement creates a hydrophobic interface and hydrogen-bonding network that likely stabilizes interdomain interactions, potentially modulating protease activity through conformational coupling. The β3 insertion may sterically occlude the catalytic site in the inactive state, suggesting a regulatory mechanism distinct from other PDZ-proteases like CtpB, where the PDZ and SPD domains are spatially separated. This structural divergence implies that SpoIVB’s PDZ domain could act as an intramolecular scaffold, with future studies focusing on mutagenesis and substrate-complex structures to elucidate how interdomain dynamics govern σ^K^ activation. Additionally, the serine protease domain of SpoIVB exhibited a distinct conformation compared to other known PDZ-proteases, with a unique spatial arrangement of the catalytic triad. The positioning of His236 and Ala378 suggests that Asp363 may not be part of the catalytic triad, implying that Thr393 might play a more prominent role in catalysis. These structural deviations could be critical for understanding the substrate specificity and regulatory mechanisms of SpoIVB.

The crystal structure also revealed an interesting interaction between the N-terminal of one SpoIVB_101-426-S378A_ molecule and the PDZ-binding motif of another molecule, suggesting that interactions between PDZ domains might involve more than just the canonical binding motif. Although the observed interaction might be artifactual, it raises the possibility that SpoIVB’s N-terminal region collaborates with its PDZ domain to engage substrates or regulators *in vivo*.

Although we successfully solved the crystal structure of SpoIVB_101-426-S378A_, other constructs, including the full-length protein and SpoIVB_75-426_, failed to yield high-quality crystals. This may be due to inherent structural flexibility or other conformational constraints of these variants, which hinder their ability to form ordered crystals. As a result, the structural information is limited to the truncated variant, and further efforts to improve crystallization conditions or use alternative techniques (such as cryo-electron microscopy) may be required for a more complete structural analysis.

### Conclusion

Overall, our study comprehensively analyzes the recombinant SpoIVB variants and their structural properties. The findings emphasize the role of the S378A mutation in altering the physical properties of SpoIVB, highlighting the structural flexibility and unique features of its PDZ and serine protease domains. The combination of crystallographic and solution-based techniques has yielded valuable insights into the conformational dynamics of SpoIVB and its potential to interact with other molecules. However, the limitations noted above indicate that further research is necessary to fully elucidate the biological function of SpoIVB and address the remaining gaps in our understanding of its structure-function relationship.

## Data Availability

The atomic coordinate and structure factor of SpoIVB_101-426-S378A_ have been deposited in the PDB under the accession code 9LNF.

## SUPPLEMENTAL MATERIAL

Supplemental material spectrum.00398-25-s0001.docx Tables S1 and S2; Fig. S1 to S5.

ASM does not own the copyrights to Supplemental Material that may be linked to, or accessed through, an article. The authors have granted ASM a non-exclusive, world-wide license to publish the Supplemental Material files. Please contact the corresponding author directly for reuse.
