# Supplementary material for: Structural characterization and biophysical analysis of recombinant SpoIVB variants: insights into PDZ and serine protease domain interactions
Source: Microbiol Spectr. 2025 Aug 14;13(10):e00398-25. doi: 10.1128/spectrum.00398-25 (PMC12502668; doi:10.1128/spectrum.00398-25)
Supplement: Supplemental material — Tables S1 and S2; Fig. S1 to S5. [file spectrum.00398-25-s0001.docx]

Supporting information for

**Structural Characterization and Biophysical Analysis of Recombinant SpoIVB Variants: Insights into PDZ and Serine Protease Domain Interactions**

Jian Zhu^1,^*, Xu Zhang^2,^*, Xinyun Zhang^1^, Gaohui Sun^1^, Peng Xu^3^, Cai Yuan^3^, Mingdong Huang^1,#^ and Longguang Jiang^1,4,#^

^1^College of Chemistry, Fuzhou University, Fuzhou, Fujian, 350116, P.R. China

^2^School of Life Sciences, Yunnan University, Kunming, Yunnan, 650091, P.R. China

^3^College of Biological Science and Engineering, Fuzhou University, Fuzhou, Fujian, 350116, P.R. China

^4^National and Local Joint Engineering Research Center on Biopharmaceutical and Photodynamic Therapy Technologies, Fuzhou University, Fuzhou, Fujian, 350116, P.R. China

*These authors contributed equally to this work

^#^Correspondence authors: Longguang Jiang, [jianglg@fzu.edu.cn](mailto:jianglg@fzu.edu.cn); Mingdong Huang, [hmd_lab@fzu.edu.cn](mailto:hmd_lab@fzu.edu.cn)

**Table S1.** Crystallization conditions of recombinant SpoIVB_101-426-S378A_

| **Method** | Sitting-drop vapor diffusion |
| --- | --- |
| **Plate type** | 96-well plate |
| **Temperature (K)** | 298 |
| **Protein concentration (mg/mL)** | 15 |
| **Buffer composition of protein solution** | 20 mM Tris-HCl (pH7.4), 300 mM NaCl, 5% glycerin |
| **Composition of reservoir solution** | 25% w/v polyethylene glycol 3350, 0.2 M lithium sulfate, and 0.1 M HEPES pH7.50. |
| **Volume and ratio of drop** | 1 μL, 1:1 |
| **Volume of the reservoir (μL)** | 100 |

**Table S2.** SAXS sample details and parameters of SpoIVB_75-426-S378A_

|  | SpoIVB_75-426-S378A_ |
| --- | --- |
| SEC column | Superdex 200 Increase 10/300 GL |
| Loading concentration (mg/mL) | 3.7 |
| Injection volume (μL) | 100 |
| Solvent | 20 mM Tris-HCl (pH7.4), 300 mM NaCl, 5% glycerin |
| **Guinier analysis** | |
| qR_g_ Max | 1.298 |
| *R_g_* (Å) | 29.76±0.18 |
| **GNOM** | |
| *R_g_* (Å) | 29.76 |
| I/(0) (cm^-1^) | 127.1 |
| D_max_ (Å) | 161 |
| *q* range (Å^-1^) | 0.042-0.397 |


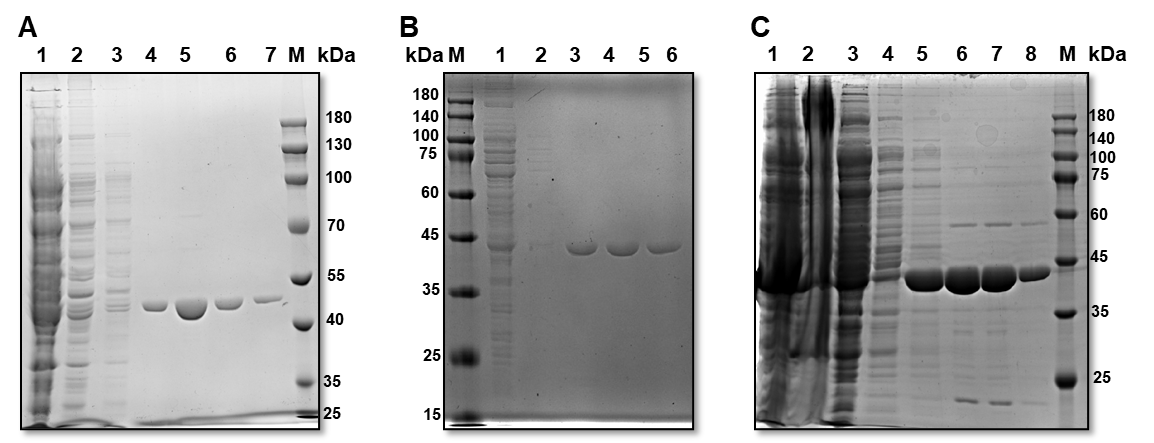


**Figure S1.** Recombinant expressions and purifications of SpoIVB_75-426_, SpoIVB_75-426-S378A_, and SpoIVB_101-426-S378A_ were confirmed by 15% SDS-PAGE. (A) Expression and purification of SpoIVB_75-426_. M: Marker, 1: cell lysis; 2. fraction from Ni-NTA column after wash with denaturing binding buffer; 3. balance buffer containing 50 mM imidazole; 4-7: SpoIVB_75-426_ eluted with elution buffer. (B) Expression and purification of SpoIVB_75-426-S378A_. M: Marker, 1: cell lysis; 2: fraction from Ni-NTA column after wash with equilibrium binding buffer; 3-6: SpoIVB_75-426-S378A_ eluted with elution buffer. (C) Expression and purification of SpoIVB_101-426-S378A_. M: Marker, 1: cell lysis; 2-3. fraction from Ni-NTA column after wash with denaturing binding buffer; 4. balance buffer containing 50 mM imidazole; 5-8: SpoIVB_101-426-S378A_ eluted with elution buffer.


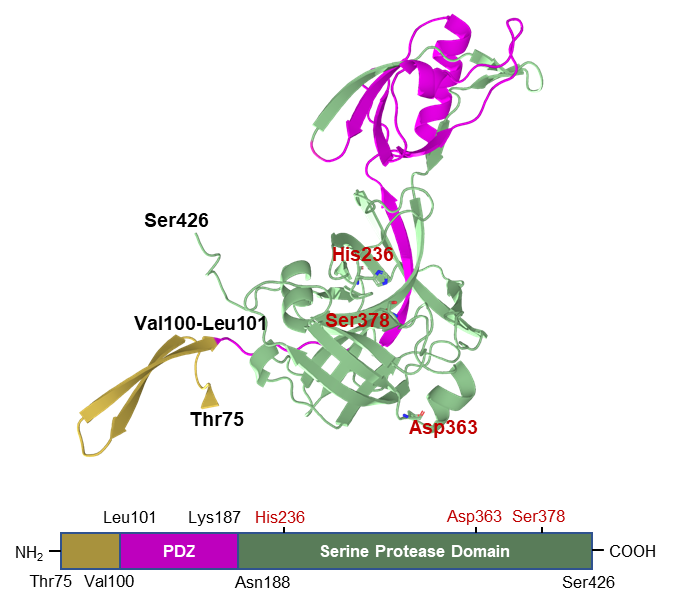


**Figure S2.** The predicted structure of SpoIVB_75-426_ includes the N-terminal part (Thr75-Val100), the PDZ domain (Leu101-Lys187), and the serine protease domain (Asn188-Ser426). The catalytic triad residues (His236-Asp363-Ser378) are shown in the stick.


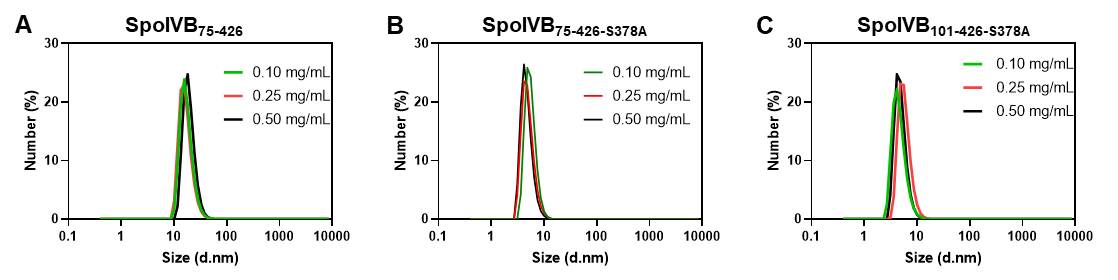


**Figure S3.** The size distributions of SpoIVB variants at different concentrations are consistent and show a single species.


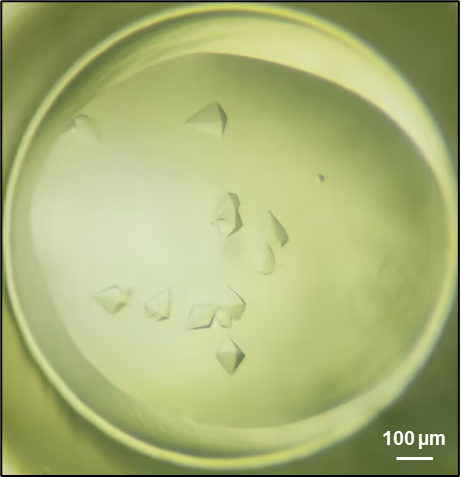


**Figure S4**. The crystals of SpoIVB_101-426-S378A_ are grown at 25% PEG 3350, 0.2 M lithium sulfate, and 0.1 M HEPES pH 7.5.


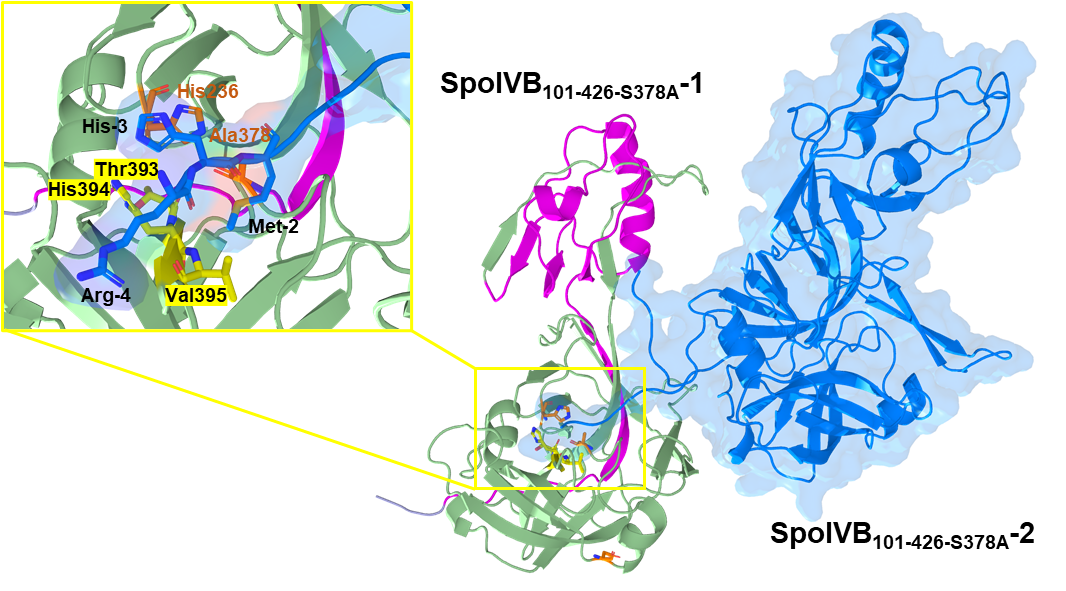


**Figure S5**. Crystal packing reveals a symmetry-related interaction between the N-terminal before the PDZ domain of one symmetrical SpoIVB_101-426-S378A_ (molecule 2) and the PDZ-binding motif (Thr393-His394-Val395) of another SpoIVB_101-426-S378A_ (molecule 1).
